# Consecutive Short-Scan CT for Geological Structure Analog Models with Large Size on *In-Situ* Stage

**DOI:** 10.1371/journal.pone.0161358

**Published:** 2016-08-18

**Authors:** Min Yang, Wen Zhang, Xiaojun Wu, Dongtao Wei, Yixin Zhao, Gang Zhao, Xu Han, Shunli Zhang

**Affiliations:** 1 School of Mechanical Engineering and Automation, Beijing University of Aeronautics and Astronautics, Beijing 100191, China; 2 Department of Earth Sciences and Institute of Energy Sciences, Nanjing University, Nanjing 210093, China; 3 Reservoir Description Key Laboratory CNPC, Lanzhou 730000, China; 4 College of Resources and Safety Engineering, China University of Mining and Technology, Beijing 100083, China; 5 School of Information Science and Technology, Northwest University, Xi’an 710127, China; Shanxi University, CHINA

## Abstract

For the analysis of interior geometry and property changes of a large-sized analog model during a loading or other medium (water or oil) injection process with a non-destructive way, a consecutive X-ray computed tomography (XCT) short-scan method is developed to realize an in-situ tomography imaging. With this method, the X-ray tube and detector rotate 270° around the center of the guide rail synchronously by switching positive and negative directions alternately on the way of translation until all the needed cross-sectional slices are obtained. Compared with traditional industrial XCTs, this method well solves the winding problems of high voltage cables and oil cooling service pipes during the course of rotation, also promotes the convenience of the installation of high voltage generator and cooling system. Furthermore, hardware costs are also significantly decreased. This kind of scanner has higher spatial resolution and penetrating ability than medical XCTs. To obtain an effective sinogram which matches rotation angles accurately, a structural similarity based method is applied to elimination of invalid projection data which do not contribute to the image reconstruction. Finally, on the basis of geometrical symmetry property of fan-beam CT scanning, a whole sinogram filling a full 360° range is produced and a standard filtered back-projection (FBP) algorithm is performed to reconstruct artifacts-free images.

## Introduction

Structure simulation experiment is a recognized approach to disclose and interpret the relation between imposed boundary conditions and resulting structures [1–3]. As the structure information carrier to observe and analyze, analog models have long been popular for simulating geological structure evolution or deformation for several decades. To achieve a more complete analysis of the spatial and temporal evolution of structures in scaled analogue experiments on all appropriate scales, especially on an in-situ stage, a fast and non-destructive imaging approach is necessary [4–7]. Since the late 1980s, the internal structure and kinematic evolution of analog models have been analyzed by X-ray computed tomography (XCT) scanning techniques originally developed for medical purposes [8–9]. On the basis of the varying attenuation of X-rays by different materials, CT technique produces cross-sectional slices of an analog model and further generates a volume data to visualize three dimensional (3D) interior geometries and properties of a model without destroying it, with more powerful XCT techniques such as spiral or helical scanners are put into use [10–14].

Among the reported researches about scanning of analog models, most are implemented on medical CT scanners. For example, Guido Schreurs et al. applied medical spiral XCT to 3D evolution of brittle viscous analog models from the initial undeformed stage to the final deformed stage, which opens new and exciting perspectives for a complete four-dimensional analysis (3-dimensional through time) of analog models and is of special importance when investigating complex structural settings [15]. Adam et al. integrated CT and digital volume correlation techniques to reveal the 3D structure and kinematic evolution of complex deformation structures in scaled analogue experiments, and further to quantify the 3D spatial and temporal strain patterns inside analogue experiments [16]. Similarly, the CT scanners used by Adam are medical CT systems (Siemens Sensation 64 and Siemens Emotion 6). It is generally known that the radiation dose is the major concern in medical XCT techniques for safety reasons [17]. Thus, the reduction of radiation dose and a decrease of the acquisition time should be the first priority, but less efficient for CT scanning of geological structure analog models with large size because the X-ray energy of medical XCTs is basically between 40~140keV for diagnostic usages, and the CT image matrix is basically 512×512 with special resolution of about 0.4mm. While scanning an analog model with larger size, much higher X-ray energy is required to penetrate it, otherwise cupping artifacts induced by beam hardening effect will be a major disturbing factor that covers the true data in the central part of CT images [18–22]. Meanwhile, much higher spatial resolution is equally important to identify more detailed information about the deformation and layered granular materials. Obviously medical XCT techniques cannot accomplish this task.

**Fig 1:**
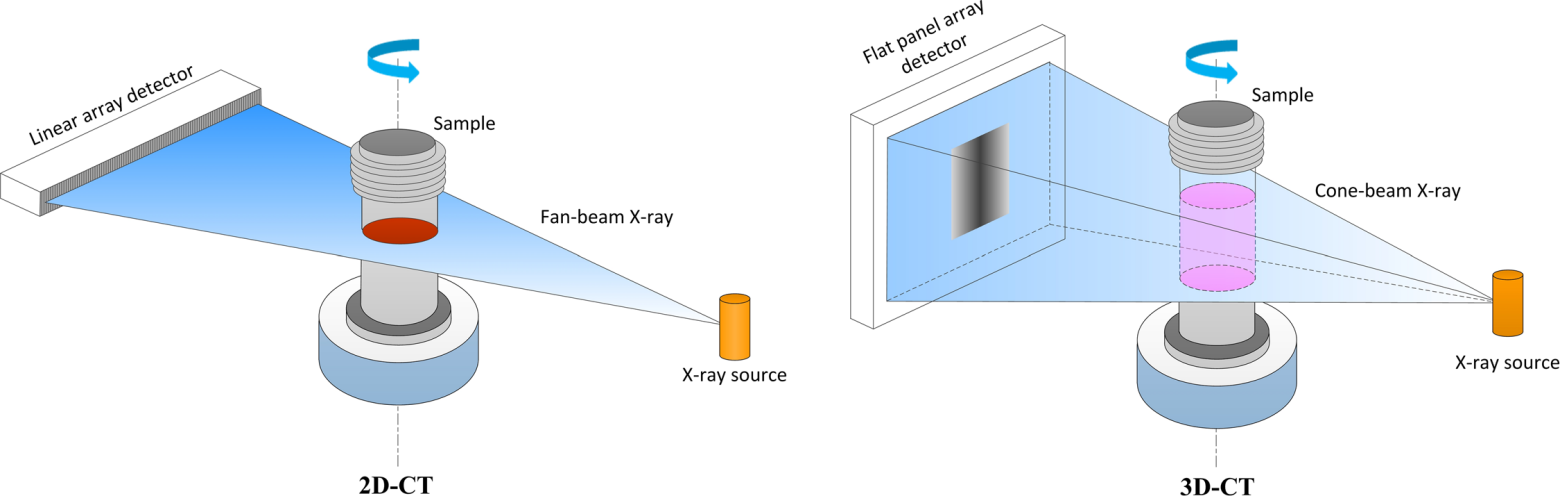
Illustration of the scanning means of IXCTs.

Compared with medical XCT scanners, industrial X-ray computed tomography (IXCT) scanners are equipped with higher energy X-ray generator and detector with smaller detecting units, and the X-ray energy and special resolution can reach 450 keV and 50μm or less, respectively, making IXCT technique become an effective approach to accomplish the scanning of analog models with large size. Fig 1 illustrates the scanning ways of typical 2&3-dimensional IXCTs. The X-ray source and the detector remain stationary with respect each other while the object rotates around a fixed center within 360 degrees. The X-ray beams (fan-beam or cone-beam) penetrating the sample at different imaging views are captured and converted to digital signals (projections) by the detector (linear array detector or flat panel array detector). Then making use of all projections, cross-sectional slices of the sample are produced by corresponding reconstruction algorithms [23–24].

**Fig 2:**
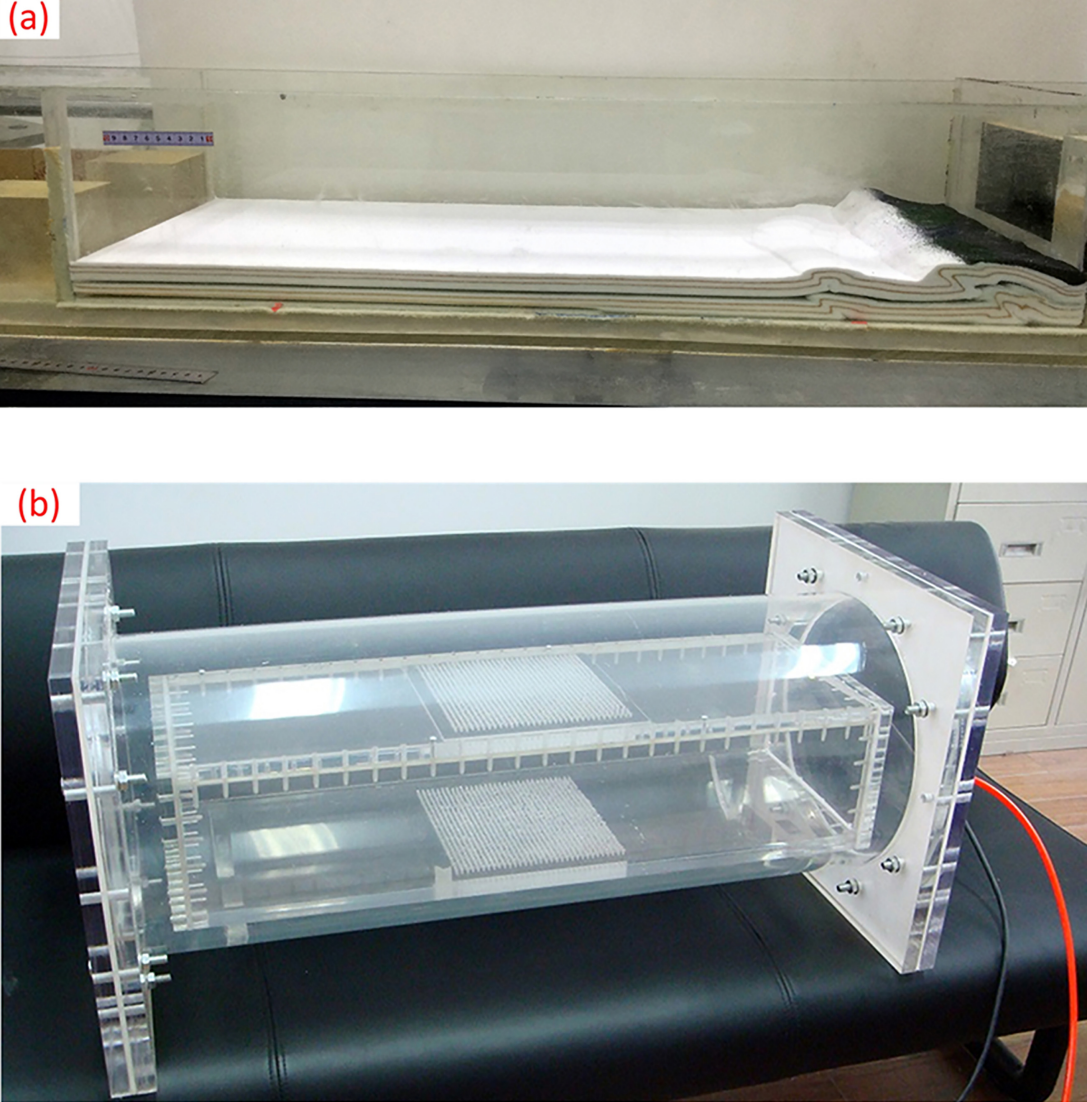
(a) An analog model, (b) A PMMA cylindrical container.

For our research here, the analog model is composed of several sand layers with different graininess and its size is about 320×120×1000 mm^3^. The analog model is closed in a PMMA cylindrical container with high inner atmospheric pressure (see in Fig 2). Driven by a hydraulic unit, external squeeze pressures are imposed on a mobile wall to produce shortening of the model and meanwhile liquid (oil and water) is sometimes injected into the model. Such a large analog model combined with external driving units requires an in-situ CT-scanning, i.e., the analog model keeps in-situ state while the X-ray tube and the detector rotate around it synchronously [25–26]. Although medical helical XCT technique is a seemingly feasible approach, installation of the model and external driving units, penetrating ability of the X-rays and spatial resolution are considerable difficulties. And, apparently, the existing IXCT scanners cannot accomplish this analogue experiment because their sample stage is rotational during a scanning.

In this paper, to reduce the hardware costs and design difficulties of driving the X-ray tube and detector to rotate a whole helical trajectory synchronously, we develop a consecutive short-scan CT method for geological structure analog models with large size on in-situ stage. In this method, the X-ray tube and detector rotate 270° by switching positive and negative directions alternately on the way of translation, which well solves the winding problem of high voltage cables and oil cooling service pipes. Also the installation of high voltage generator and cooling system becomes easier. Then, a structural similarity based method is applied to elimination of the invalid projection data. By using the data symmetry property of fan-beam CT, a whole sinogram filling a full 360° range is produced. Finally, a standard filtered back-projection (FBP) is performed and artifacts-free images are obtained.

## Method

### Consecutive CT short-scan trajectory

**Fig 3:**
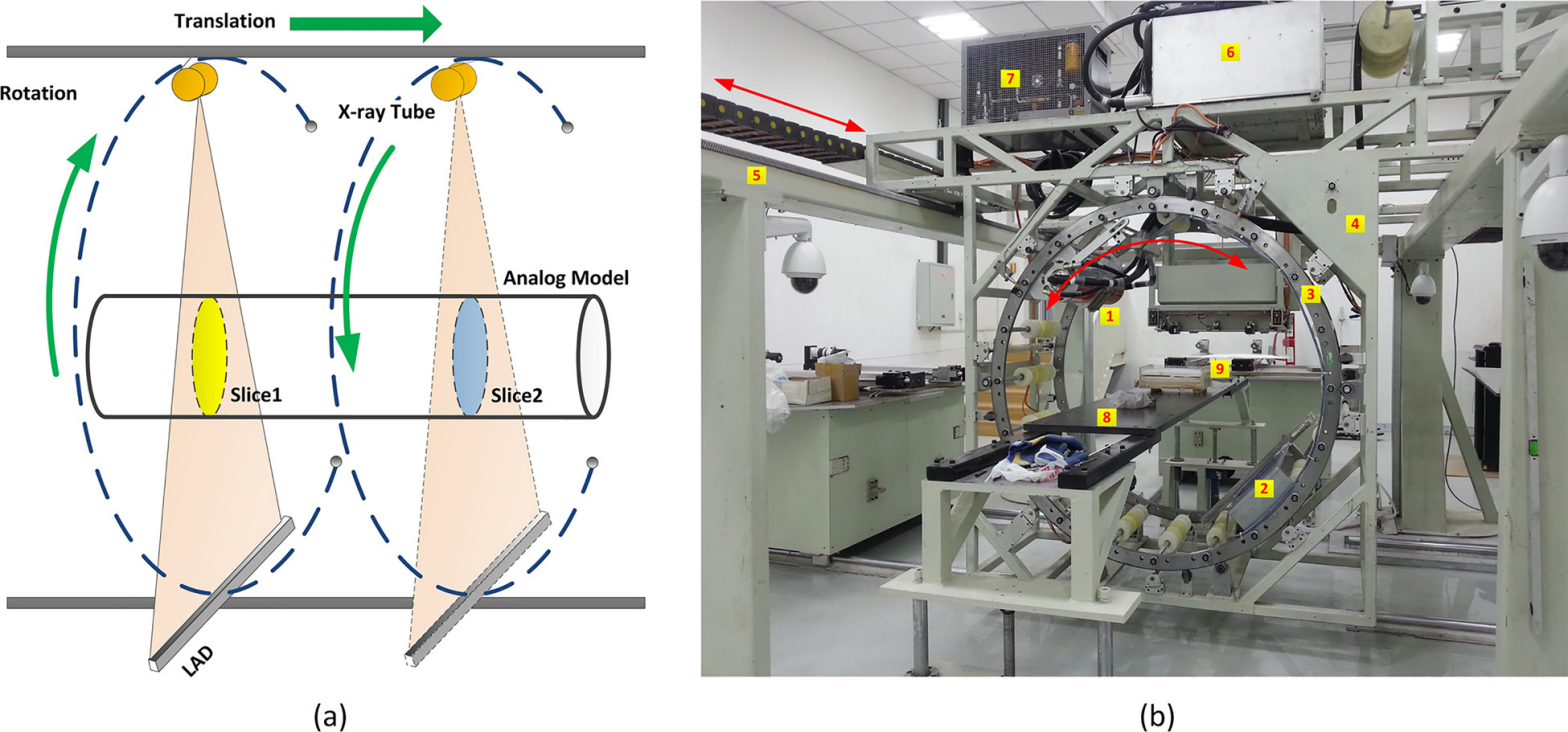
(a) Illustration of the consecutive short-scan CT trajectory; (b) Consecutive short-scan CT system in the integration: 1. X-ray tube; 2. Linear array detector; 3. Circular guide rail; 4. Suspended frame; 5. Supporting truss girder; 6. High-voltage generator; 7. Cooling system; 8. Sample stage; 9.Analog model.

For the CT scanning system we developed, the initial cone-beam generated by an X-ray tube is collimated to a fan-beam, and a linear array detector (LAD) with high dynamic range and spatial resolution matches the fan-beam to constitute a 2-dimentional CT scanning setup. Different from a full 360°-circle scanning trajectory in traditional IXCT and a whole helical scanning trajectory in medical XCT, we employ a consecutive short-scan way as shown in Fig 3A. The X-ray tube and the LAD are installed on a circular guide rail. For a cross-sectional slice acquisition, the X-ray tube and LAD rotate 270° around the center of the guide rail synchronously, then move a small step along the lengthwise direction of the analog model and rotate 270° in the opposite direction to accomplish the next cross-sectional slice acquisition. This kind of scanning cycle repeats itself until all the needed cross-sectional slices are obtained.

Fig 3B is our consecutive short-scan CT system in the integration, the rotating components mainly include a circle guide rail, a LAD and an X-ray tube. They are mounted on a suspended frame hanged on a supporting truss girder. The high-voltage generator and cooling system are fixed on the top of the suspended frame and always keep stationary during a scanning. Linear sliders connect the suspended frame and the supporting truss girder, enabling the suspended frame to move backward and forward to change the position of the cross-sectional slice. In order to ensure a steady rotation of the X-ray tube and LAD, a counter weight is combined with the LAD to minimize the moment of inertia of the guide rail during a rotation. The installation must guarantee the beam-line connecting the X-ray focus and the midpoint of the LAD to pass the center of the circular guide rail. The circular guide rail is a double gear rings with high precision, and is driven by two synchronous motors to realize the rotation movements. For such a scanning setup, if the X-ray tube and the LAD rotate a whole circle, the winding of the high voltage cables and oil cooling service pipes will be inevitable definitely. We hence choose an unclosed circle trajectory with alternatively varying directions to make the cables and pipes wind and unwind in turn smoothly around the circular guide rail. The feed mechanism is dedicatedly designed to ensure that the tension and winding of the cables and pipes will not impact their performance and threaten the safety.

When starting an in-situ CT scan, the analog model with loads and injected fluids is placed on the sample platform which is located nearly at the center of the circular guide rail. The host PC sends commands to the motion controller to drive the X-ray tube and LAD to move simultaneously to acquire a whole digital radiography projection of the analog model, or to drive the X-ray tube and LAD to rotate synchronously to acquire the raw sinogram of a cross-sectional slice. During a 270° rotation, the LAD receives X-ray photons penetrating the analog model and converts them to a row of digital signals. All the row data from different projection views constitute a two-dimensional matrix, i.e., a sinogram. After pre-processing on the raw sinogram, a GPU-accelerated FBP reconstruction is performed and artifacts-free cross-sectional images are obtained.

### Elimination of the invalid data in a raw sinogram

**Fig 4:**
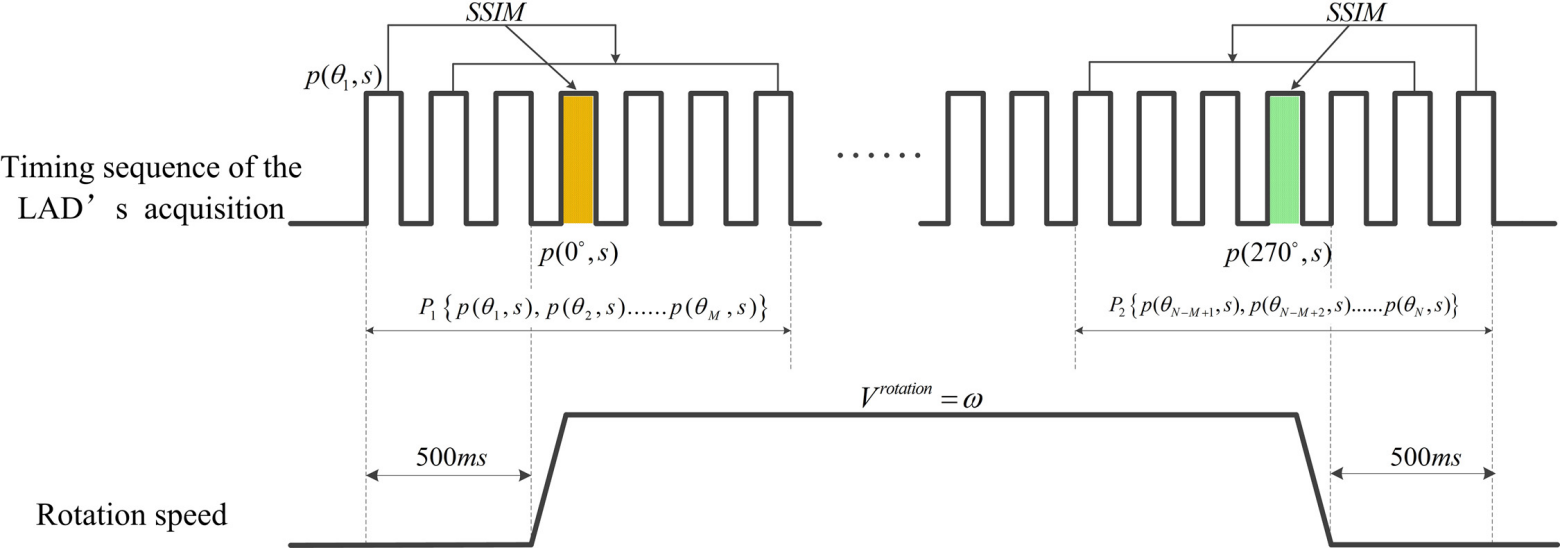
Illustration of elimination of the invalid data in a raw sinogram.

In order to acquire the effective projections among a 270° rotation, as shown in Fig 4, the LAD is first triggered to collect projections 500ms ahead of the simultaneous rotation of the X-ray tube and LAD. For our research, the working integration time of LAD is 50ms and the rotating speed is 6° / *s*. All the collected projection data are automatically transmitted to a RAM or hard disk of the host PC through a LAN cable. When the rotation reaches 270° position, the host PC sends commands to the motor to terminate the rotation, and 500ms later, the LAD finishes data collection. Obviously, such a way can make the sinogram spans a whole 270° range, but because of the 500ms’s extra data collection of the LAD at the startup stage and the ending stage, respectively, some invalid data are created and they occupy several rows on both ends of a raw sinogram. This kind of invalid data do not contribute to the image reconstruction, and even bring out artifacts in the reconstructed images instead. Thus, they must be identified accurately and eliminated before reconstruction. Theoretically, the length of the invalid data can be calculated according to the integration time and rotating speed. However, due to the acceleration and deceleration of the motor at startup and ending stages, response lag of the control terminals to the host PC, a part of the invalid data are uneven-distributed, resulting in the length of the invalid data is always unstable. Therefore, a more flexible and adaptive method is necessary to identify the invalid data.

As we know, the invalid data are collected at a non-rotational stage, thus, each projection during the starting 500ms period should be the same if random noise and X-ray intensity fluctuation are ignored, because they are from the same projection view. Similarly, this is true for the ending 500ms period. Here, we develop a structural similarity (SSIM) based method to identify the invalid data. SSIM is an index for measuring the similarity between an initial or distortion-free image and processed image [27–28]. It defines structure information independent of brightness and contrast, and reflects the attributes of the objects more objectively. Especially, SSIM-based method is not sensitive to image noise. Thus, it is widely used in the similarity comparison between two images with simple operation and high accuracy. The calculation formula of SSIM can be written as:

$$\text{SSIM}(\text{A},\text{B})=\frac{\left(2\mu_{A}\mu_{B}+C_{1})(2\sigma_{AB}+C_{2}\right)}{\left(\mu_{A}^{2}+\mu_{B}^{2}+C_{1})(\sigma_{A}^{2}+\sigma_{B}^{2}+C_{2}\right)}$$ (1)

Where A is the reference projection and B is an arbitrary projection to process. *μ*_*A*_, *μ*_*B*_ are the mean value of A and B. *σ*_*A*_, *σ*_*B*_ are the standard deviations of A and B. *σ*_*AB*_ is the covariance between A and B. *C*_1_, *C*_2_ are constants with small value, which are used to ensure the denominator is nonzero. The values of *C*_1_ and *C*_2_ are proved to be non-sensitive to the SSIM results and can be set in a wide range with small values. Here we set *C*_1_ = *C*_2_ = 0.02. A larger SSIM indicates a higher similarity between an image couple. A raw sinogram is defined as *p*(*θ*,*s*), where *θ* is the rotating angle, *s* is the detecting unit coordinate of the LAD. According to the proposed method, *M* rows are abstracted from the start and end of *p*(*θ*,*s*), respectively. And the two data sets are expressed as *P*_1_{*p*(*θ*_1_,*s*),*p*(*θ*_2_,*s*)……*p*(*θ*_*M*_,*s*)} and *P*_2_{*p*(*θ*_*N−M*+1_,*s*),*p*(*θ*_*N−M*+2_,*s*)……*p*(*θ*_*N*_,*s*)}, where *N* is the height of *p*(*θ*,*s*). *M* is set by the following empirical method:

$$M>\frac{Non\text{-}rotationalTime(i.e.,500\;ms)}{IntegrationTime(i.e.,50\;ms)}+offset.$$ (2)

Where *offset* is used to make *P*_1_ and *P*_2_ contain some projections from the rotational stage and its suggested value is 5-10. Then we calculate each SSIM coefficient between the first row *p*(*θ*_1_,*s*) and other rows in *P*_1_. Observing the changes of SSIMs, we can find that from the beginning, SSIM almost remains the same value very close to 1 for a distance where the un-rotational stage lies, until a distinct drop appears and then keeps changing on a lower level, which indicates that the projections enter the rotational stage. So we have the reason to believe that the inflection point of SSIMs’ variation is just the position of 0°-projection, i.e., *p*(0°,*s*). In the same way, each SSIM coefficient between the last row *p*(*θ*_*N*_,*s*) and other rows in *P*_2_ is calculated and inflection point of their distribution is the 270°-projection, i.e., *p*(270°,*s*). For the location of the inflection point, we adopt the maximum gradient algorithm to search the distinct drop point of the distribution of SSIMs [29–33].

InflectionPoint={SSIM(θ)|SSIM=arg(max(|∇SSIM(θ)|))} (3)

After the two inflection points at 0° and 270° are found, the data outside them are eliminated and the effective projections accurately spanning 0°∼270° range are finally obtained for accurate image reconstruction.

### Short-scan CT reconstruction algorithm

**Fig 5:**
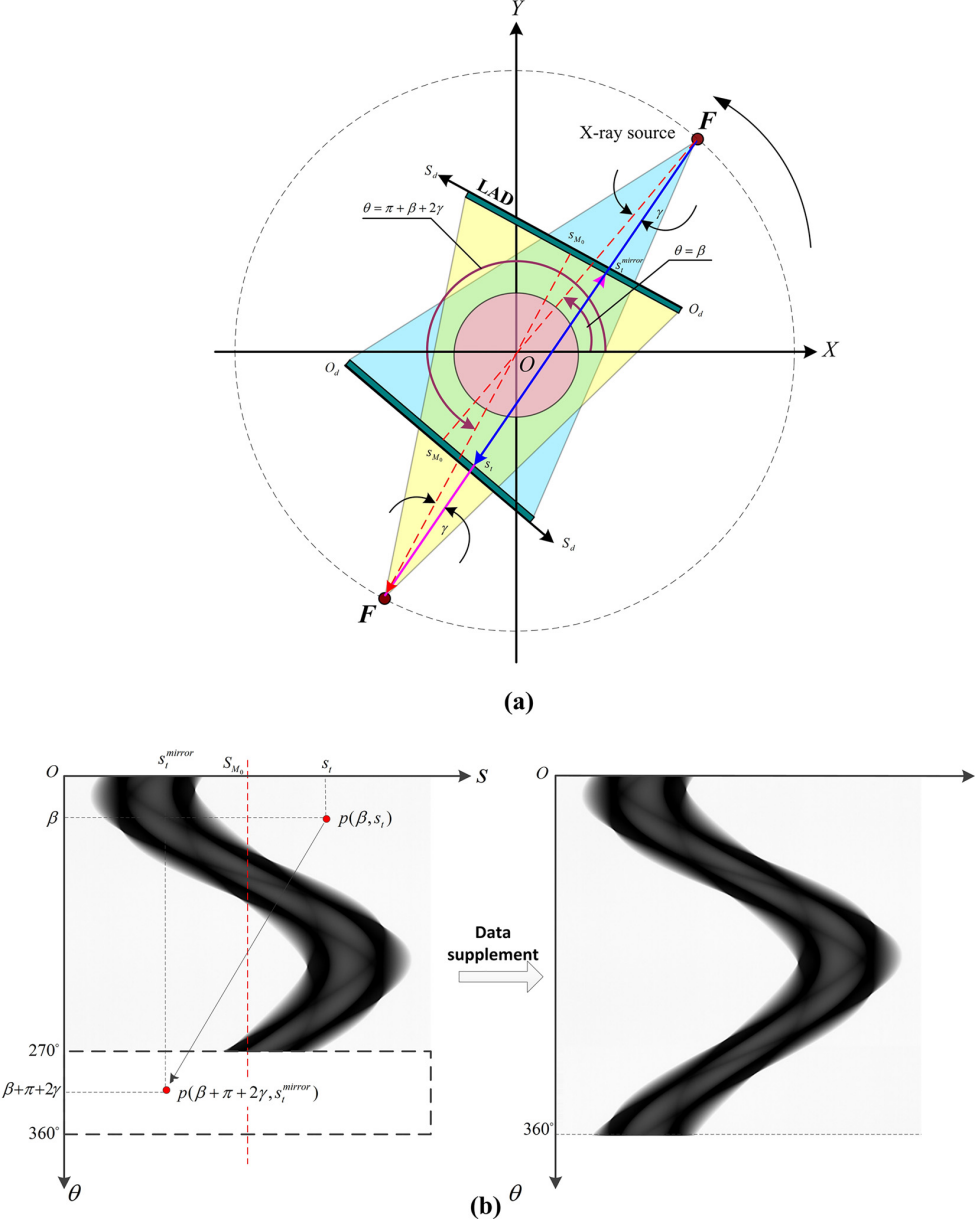
(a) Illustration of geometrical symmetry of fan-beam CT; (b) Illustration of data supplement to achieve a complete sinogram.

For a traditional fan-beam CT system, the X-ray source and the detector rotate 360° around a fixed center synchronously while the object remain stationary as shown in Fig 5A. The ray connecting the focus and the detecting unit *s*_*t*_ is labeled by the angle (named *γ*) between it and the central X-ray. The central X-ray is the beam perpendicular to the LAD and passing the center of rotation. The projection address of the central X-ray, namely the projection address of the center of rotation is named as $s_{M_{0}}$. At rotation angle *θ* = *β*, *β* ∈ [0 2*π*], the ray *γ* passes the scanned slice and reaches the detecting unit *s*_*t*_, producing the projection value of *p*(*β*,*s*_*t*_). Similarly at rotation angle *θ* = *β*+*π*+2*γ*, the projection address and value of ray *γ* are $s_{t}^{mirror}$ and $p(\beta +\pi +2\gamma ,s_{t}^{mirror})$, respectively. From the geometrical configuration of the fan-beam scanning, we could find that the ray *γ* penetrates the scanned slice along a same path at *β* and *β*+*π*+2*γ* rotating angles, so it can be deduced that point *s*_*t*_ and $s_{t}^{mirror}$ are symmetrical about point $s_{M_{0}}$ and the projection values at this two points are equal. Namely,

$$s_{t}=2s_{M_{0}}-s_{t}^{mirror}$$

$$p(\beta ,s_{t})=p(\beta +\pi +2\gamma ,s_{t}^{mirror}),\begin{array}{cccc} & & \beta \in [0 & 2\pi ] \end{array}$$

$$\gamma =\arctan(\frac{\left|s_{t}-s_{M_{0}}\right|}{D})$$ (4)

Where, *D* is the distance from the X-ray source to the LAD.

From Eq (4), we can find that $S_{M_{0}}$ and *D* are the dominating parameters for the location of equivalent pixels, and then affect the reconstruction accuracy. For our experiments, we first locate the center of rotation by image cross correlation method [34–35]. According to this method, the raw sinogram is averaged to a data set with regular symmetric shape. Then the data set is flipped along the direction of row to create a new one. And cross correlation operation is applied to these two data sets and finally the position of $S_{M_{0}}$ is determined by locating the peak value of the cross correlation function. For the measurement of the distance from the X-ray source to the LAD, we first acquire projection images of a double-circled object at two imaging positions. Then through auto-correlation operation and solving an equation, the value of *D* is finally obtained [36].

Upon the above analysis, we find that when a sample is scanned around a whole 360°, redundant data will be produced for the sake of geometrical symmetry of fan-beam CT. Thus, on the basis of this fact, so long as the rotation angle is larger than 180° + 2*γ*_*m*_ and less than 360°, i.e., through a short scanning, we still can get a whole sinogram filling a full 360° range according to Eq 4. Here 2*γ*_*m*_ is the fan-beam angle. As the 270°-short scanning way we adopt, the raw sinogram is a short one and if being used for reconstruction without any pre-processing, artifacts will be resulted in. Actually, as shown in Fig 5B, the missed data in [270° 360°] projection views can be retrieved upon the already collected projections by LAD in [0° 270°] views. For example, *p*(290°,*s*_*t*_) is a missed projection data, on the basis of the geometrical symmetry property of fan-beam CT, an unique equivalent pixel $p(\theta_{}^{mirr},s_{t}^{mirr})$ must exist on the raw sinogram and has the same value with *p*(290°,*s*_*t*_) according to Eq (4). Thus, we can get: *θ*^*mirr*^ = 290° + 180° + 2*γ* = 110° + 2*γ* < 270°, demonstrating that $p(\theta_{}^{mirr},s_{t}^{mirr})$ is an already collected projection data. After a complete 360°-sinogram is achieved by this way, a standard FBP reconstruction is performed and artifacts-free images can be obtained.

## Results

**Fig 6:**
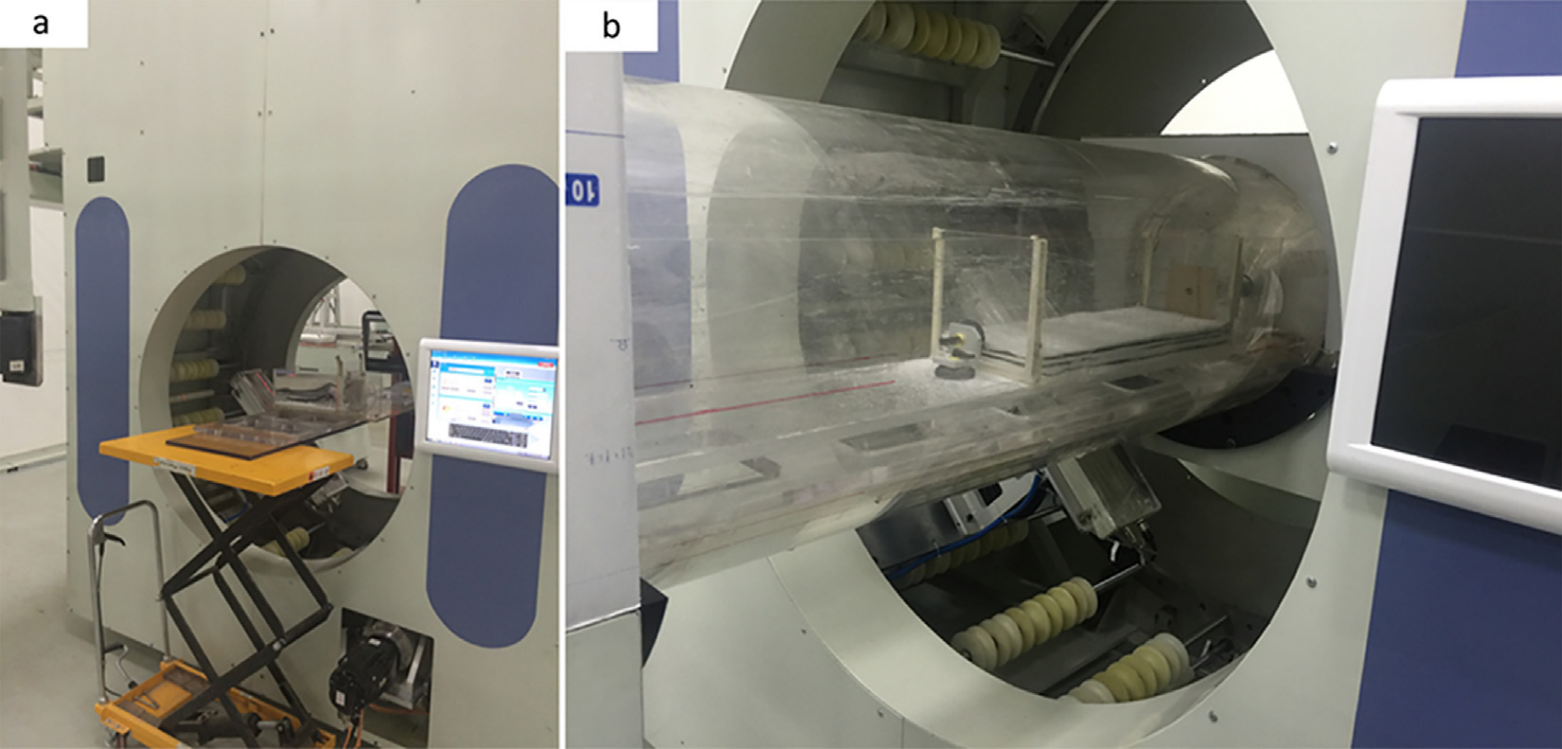
(a) The finished consecutive short-scan CT system; (b) In-situ CT scanning of an analog model in extrusion process.

**Fig 7:**
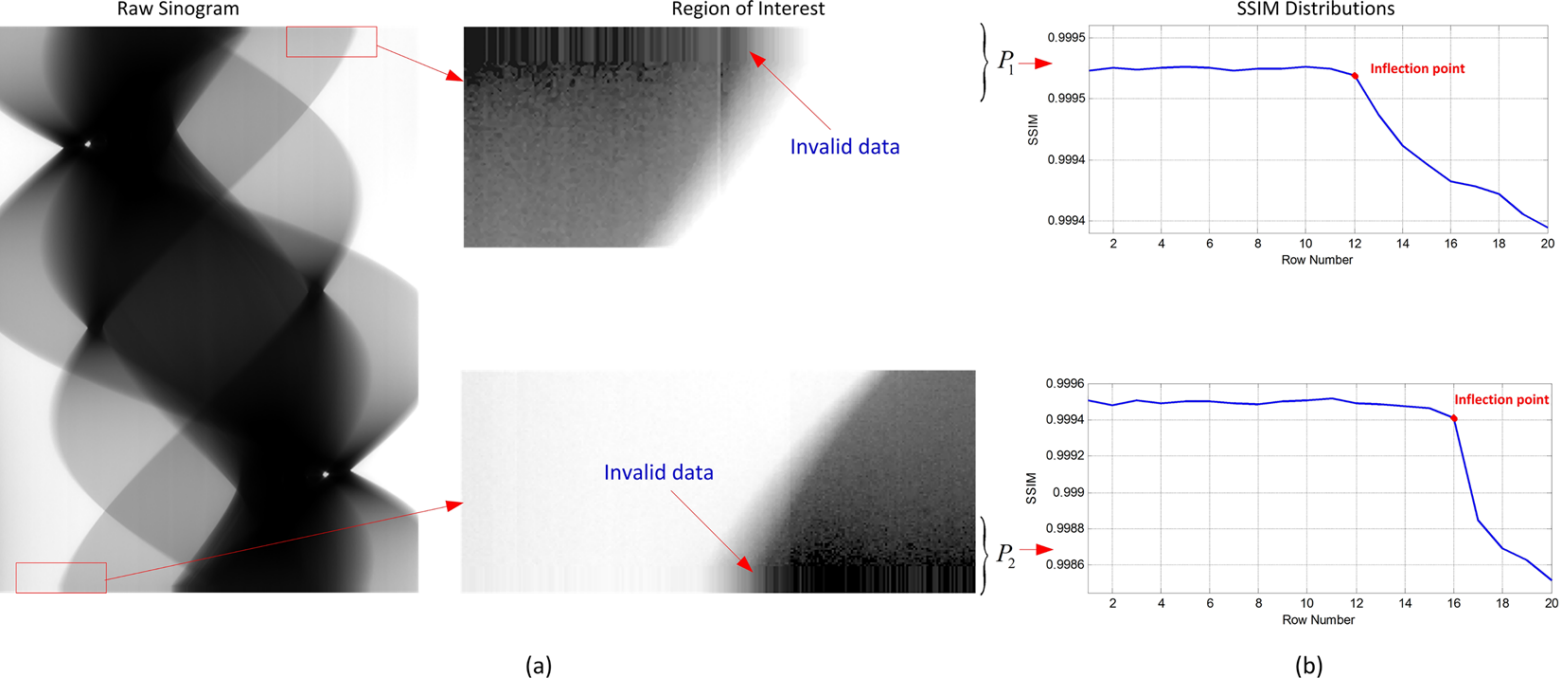
Raw sinogram and SSIM distributions. (a) A raw sinogram including invalid data at both ends, we can see that the invalid data are collected at a non-rotational stage and they are nearly the same, thus, they are strip-like on the raw sinogram; (b) SSIM distributions of dataset *P*_1_ and *P*_2_. We can find that from the beginning, SSIMs are almost close to 1 for a distance, then a distinct drop arises and SSIM goes on changing on a lower level. Thus, we have the reason to believe that the inflection points are the exact positions where 0° & 270°-projections lie.

**Fig 8:**
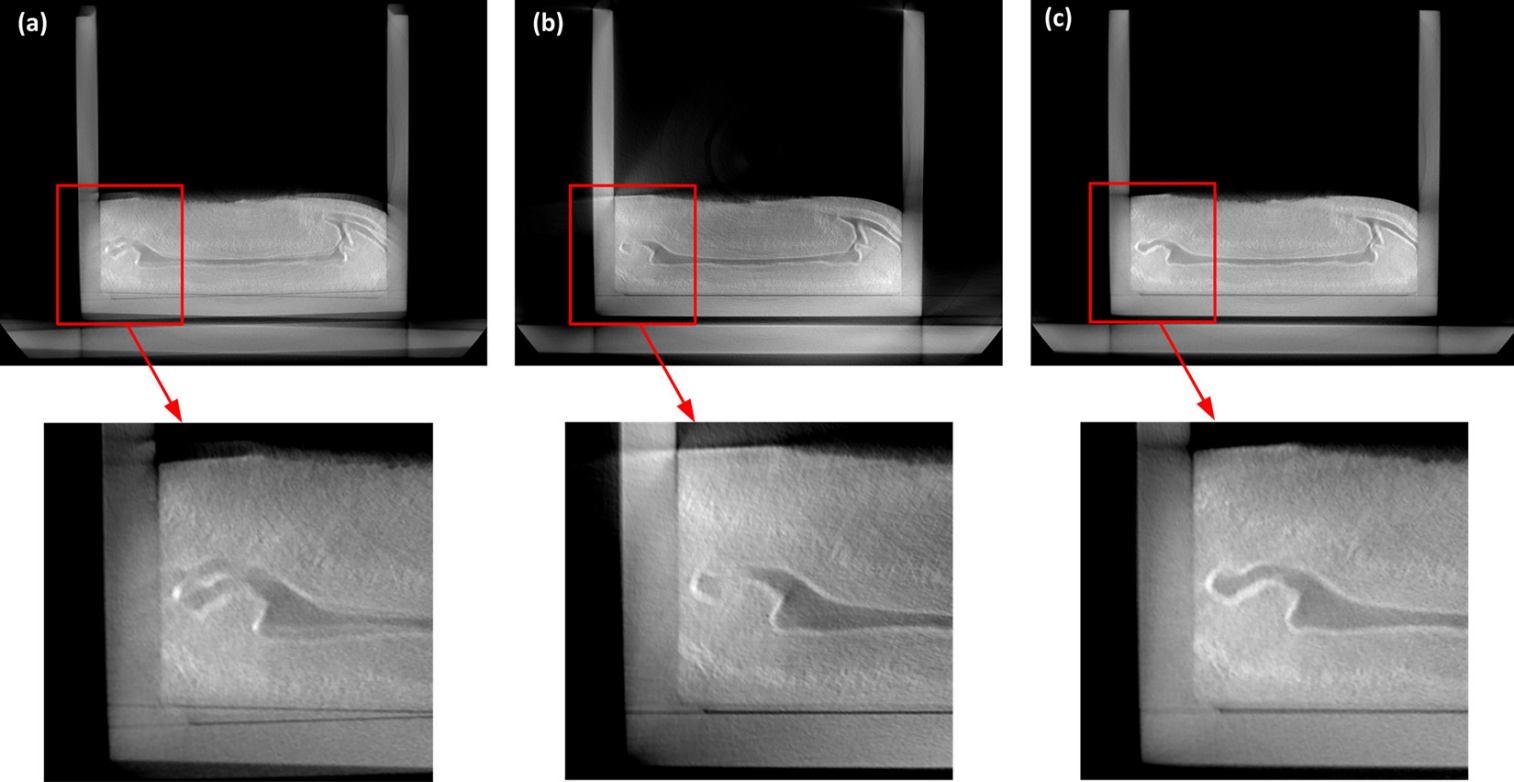
Reconstructed images by different sinograms (a) Reconstructed image by a raw sinogram, geometrical distortion artifacts are produced due to the invalid data and a non-360°-sinogram involved in the back-projection; (b) Reconstructed image by an accurate 270°-sinogram, due to the unclosed back-projection operation, some detailed textures are lost and weak string artifacts exist; (c) Artifacts-free reconstructed image by an complemented 360°-sinogram.

Fig 6 shows the finished short-scan CT system on which we performed experiments of in-situ scanning of an analog model in extrusion process. The main experimental parameters are shown in Table 1. The offset and gain corrections of the LAD are accomplished in advance to avoid dark field noise and response non-uniformity of the detecting units [37–39]. Geometrical calibrations such as focus to detector distance, center of rotation, and pixel size are also necessary to measure for an accurate image reconstruction. The prepared sandbox is closed in a PMMA cylindrical container with 3K~2Mpa inner air pressure. And while external squeeze pressures are being loaded to a mobile wall of the sandbox to produce shortening of the model, the X-ray tube and LAD rotate 270° around the center of the guide rail synchronously to accomplish scanning of one cross-sectional slice, then move 5mm along the lengthwise direction of the analog model and rotate 270° in the opposite direction to accomplish the next cross-sectional slice acquisition. The cycle repeats itself until all the needed cross-sectional slices are obtained. Fig 7A is a raw sinogram which only spans 270° range. As analyzed before, although the proposed data collection strategy ensures that a rotation scanning spans a whole 270° range, invalid data are produced at both ends of the raw sinogram inevitably. In this case, if a FBP reconstruction is performed upon the raw sinogram, obvious artifacts are produced on the reconstructed image as shown in Fig 8A, because the invalid data are involved in the back-projection and the raw sinogram is not a whole 360°-one, resulting in a mismatching between projections and rotation angles.

**Table 1:** Experimental parameters in a short CT scanning.

X-ray tube voltage	410keV
X-ray tube current	3.0mA
Pixel size	0.166mm
Length of LAD	800mm
Integration time	50ms
Rotation speed	6 degrees/s
X-ray focus to LAD distance	1310mm
Translation pitch	5mm

According to the SSIM-based method of invalid data elimination, 20 rows are extracted from the beginning of the raw sinogram to constitute a data set *P*_1_. The SSIMs between the first row and other rows in *P*_1_ are calculated and their distribution is shown in Fig 7B, where we can find that from the beginning, SSIM keeps almost close to 1 for a distance, then a distinct drop arises and then SSIM goes on changing on a lower level. By maximum gradient location algorithm, the 0°-projection is at the 12^th^ row from the beginning. Next, we extract the other data set *P*_2_ including the last 20 rows of the raw sinogram. Similarly, the SSIMs between the last row and other rows in *P*_2_ are calculated and the inflection point of SSIM’s variances is located at the 16^th^ row from the end of the raw sinogram with a total of 924 rows. Therefore, the sinogram which accurately spans 0° ∼ 270° is from the 12^th^ row to the 909^th^ row, i.e the effective sinogram for accurate reconstruction. After the above processing, an effective sinogram is obtained, but it is still a short sinogram which doesn’t fill the whole 360° range. If it is used for reconstruction directly, due to the unclosed back-projection operation, artifacts are brought out in the reconstructed image as shown in Fig 8B.

**Fig 9:**
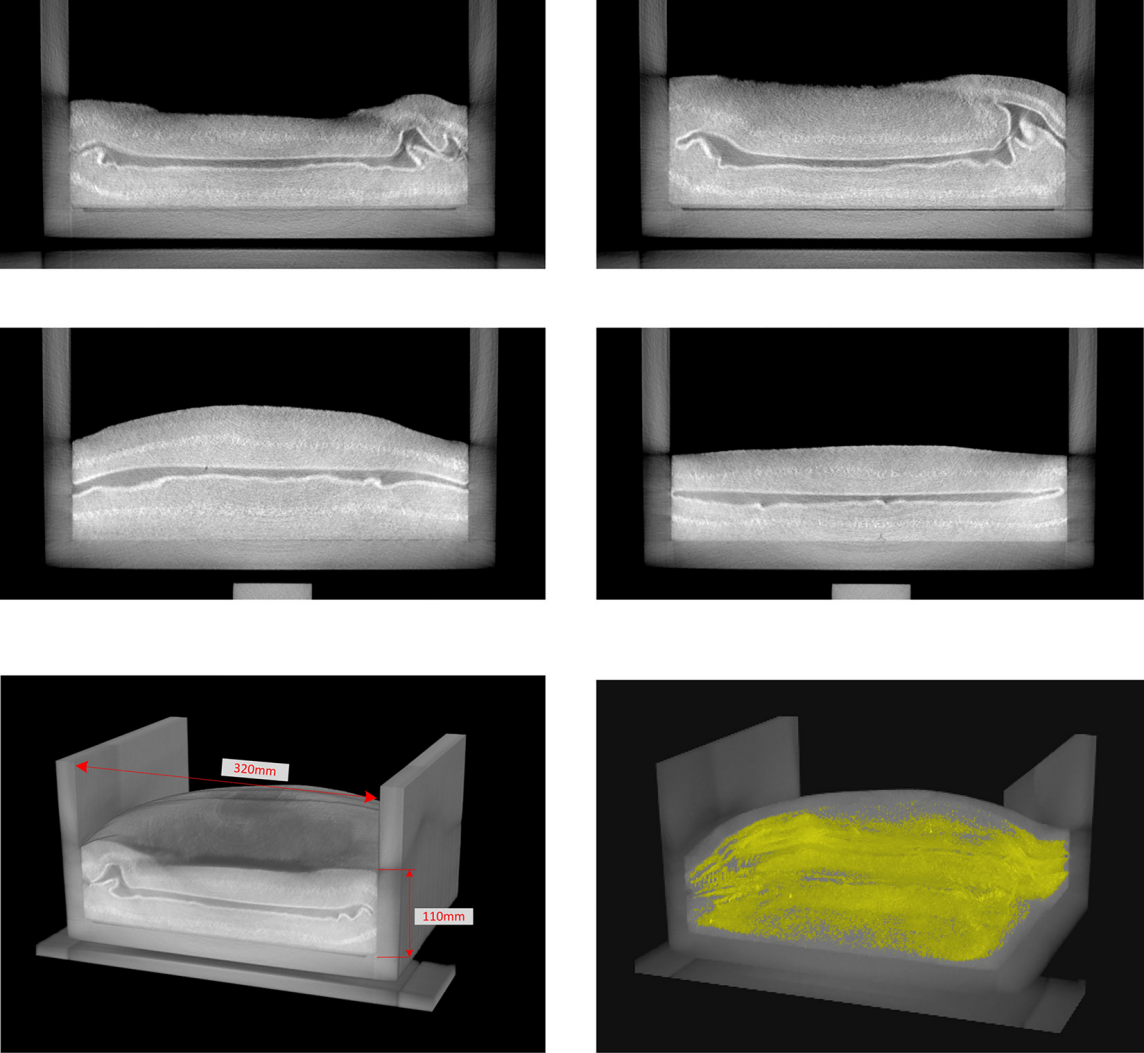
Typical cross-sectional slices and 3D virtual models of an analog model.

On the basis of the geometrical symmetry property of the fan-beam CT scanning, the missed data in [270° 360°] projection views are retrieved upon the already collected projections by LAD in [0° 270°] views. Thus, a whole sinogram which fills a full 360° range is produced for a standard FBP reconstruction. Finally, an artifacts-free image is obtained as shown in Fig 8C. In Fig 9 some typical slices are presented and two 3D virtual models are reconstructed, which well reveals the interior geometries and properties of the analog model.

## Conclusion

For the visualization of interior geometry and property changes of large-sized analog models during a loading or other medium (water or oil) injection process, the models are required to keep on an in-situ state, and the X-ray tube and the detector are required to rotate around a fixed center synchronously. Medical XCT technique is a seemingly feasible approach, but installation of the model and external driving units, the penetrating ability of the X-rays and the spatial resolution are considerable difficulties. Compared with medical XCT scanners, IXCT scanners are equipped with higher energy X-ray generator and detector with smaller detecting units, making IXCT technique an effective approach to resolve the in-situ scanning of analog models with large size. Unfortunately, the existing IXCT scanners cannot accomplish this analogue experiment because their sample stage is rotational during a scanning. For these reasons, we develop a consecutive short-scan CT method for analysis of geological structure analog models with large size on in-situ stage. In this method, the X-ray tube and LAD rotate 270° around the center of the guide rail synchronously to accomplish scanning of one cross-sectional slice, then move a short distance along the lengthwise direction of the analog model and rotate 270° in the opposite direction to accomplish the next cross-sectional slice acquisition. The cycle repeats itself until all the needed cross-sectional slices are obtained. This method solves the winding problem of high voltage cables and oil cooling service pipes well. Also the installation of high voltage generator and cooling system becomes easier. Of course, the hardware costs are also significantly decreased. In order to acquire the effective projections that accurately span a whole 270° range, we propose a data collection strategy which permits the LAD to collect data 500ms ahead of the beginning of rotation and 500ms behind the ending of rotation, which produces invalid data at both ends of the raw sinogram. A structural similarity based method is applied to elimination of the invalid data, and effective projections which well match rotation angles are obtained. Then on the basis of the geometrical symmetry property of the fan-beam CT scanning, a whole sinogram which fills a full 360° range is produced and a standard FBP algorithm is performed to realize artifacts-free images reconstruction.

Despite the encouraging results presented in this paper, there still some problems which our future research will focus on. For example, while the X-ray source works on a high tube voltage, scatter and ring artifacts will be the main factors degrading the image quality, and when the projection of an analog exceeds the length of LAD, data truncation will be caused and the reconstruction algorithm used here will not be applicable. Meanwhile, more professional and accurate interpretation to the 3D reconstruction results will continue in our recent future research report.
